# Relationship Between Sexual Activity, Contraceptive Utilization and Biopsychosocial Characteristics Among Homeless Shelter Adolescents

**DOI:** 10.7759/cureus.18128

**Published:** 2021-09-20

**Authors:** Brittney A Gaudet, Nina Liu, Allison N Kayne, Taylor L Jarvill, Cecilia Zemanek, Jeffrey M Downen, Hoonani M Cuadrado, Amy B Smith, Marna R Greenberg, Jessica L Jacoby, Joanne N Quinones

**Affiliations:** 1 Department of Emergency and Hospital Medicine, University of South Florida Morsani College of Medicine/Lehigh Valley Health Network Campus, Allentown, USA; 2 Department of Community Health and Health Studies, University of South Florida Morsani College of Medicine/Lehigh Valley Health Network Campus, Allentown, USA; 3 Street Medicine, Valley Health Partners, Allentown, USA; 4 Department of Education, University of South Florida Morsani College of Medicine/Lehigh Valley Health Network Campus, Allentown, USA; 5 Department of Emergency Medicine, University of South Florida Morsani College of Medicine/Lehigh Valley Health Network Campus, Allentown, USA; 6 Department of Obstetrics and Gynecology, University of South Florida Morsani College of Medicine/Lehigh Valley Health Network Campus, Allentown, USA

**Keywords:** homeless youth, contraception, adolescent, reproductive health, sexual activity

## Abstract

Objective*:* To determine whether biopsychosocial factors are associated with sexual activity and contraceptive utilization among homeless shelter adolescents.

Methods*: *A retrospective study of 440 adolescents at a shelter in Pennsylvania between February 2015 and September 2019 was conducted. The cohort was evaluated to determine what relationship age, gender identity, substance use, and trauma history have with sexual activity and contraceptive utilization.

Results*: *Sexual activity was significantly related to age (mean 15.8+1.4 years in sexually active vs. 14.7+1.6 years in abstinent youth, p<0.001); remote history of self-harm behavior (relative risk ratio (RR) 1.23 [95% CI 1.03-1.46]; p=0.02), history of aggressive behavior (RR 1.21 [95% CI 1.01-1.46]; p=0.04), history of trauma (RR 1.24 [95% CI 1.04-1.48]; p=0.03), and substance use (RR 2.27 [95%CI 1.86-2.77]; p<0.001). There were 55.7% sexually active females vs. 42.50% males reporting contraception use (p=0.01). After adjustment, older age and substance use remained significantly associated with sexual activity (adjusted odds ratio (AOR) 1.58 [95% CI 1.36-1.83]; p<0.001 and AOR 5.18 [95% CI 3.28-8.18]; p<0.001, respectively).

Conclusions*:* Females self-reported sexual activity using contraception more than males. After adjustment, older age and substance use were associated with sexual activity. By better understanding the impact these factors can have on contraceptive utilization, informed policy and practice interventions can be developed and implemented to help increase safe sex practices in spaces where homeless adolescents access healthcare.

## Introduction

Every year in the United States, approximately 1.6 million youth under age 18 and nearly 3.5 million young adults between the ages of 18 and 25 experience housing insecurity [1,2]. A lack of safe housing begets many challenges for this population, one of which is healthcare. Unsheltered adolescents face diverse disparities in health care access and outcomes which may be exacerbated by a lack of transportation, insurance, money, health literacy, and support systems [3]. Given the challenges in getting to and navigating the healthcare system, that is not surprising [3]. Of course, the disparities evident in this population do not exclude reproductive health behaviors and outcomes. Unhoused youth are at significantly higher risk for negative reproductive health outcomes, including sexually transmitted infections (STIs) and unplanned pregnancy, than their housed peers [4]. Some reports have estimated that unsheltered adolescents are six times more likely to become infected with Human Immunodeficiency Virus (HIV) than those with access to secure housing [2]. The risk of becoming HIV positive only increases the longer an adolescent faces homelessness [5,6]. Gaps in care access and quality exist within subsets of this population as well. Notably, unhoused persons born with female reproductive anatomy face disparities in pregnancy-related outcomes [7]. Young women facing homelessness are four to eight times more likely to become pregnant than non-homeless young women [8]. Their risk of becoming pregnant is compounded by their risk of poor ante-, intra- and post-partum outcomes [9,10]. It is important to underscore that the risk of unwanted or unplanned pregnancy is not unique to homeless women. Between 22-43% of unhoused young men indicate that they have been involved in the conception of unwanted pregnancy [11].

The issue of disparate reproductive health outcomes in this population is not a new one, but it is worsening. After nearly a decade of decline in the 2000s, the likelihood of negative reproductive health outcomes and rates of STIs in the homeless adolescent population has been increasing over the past ten years [6]. While the sheer differences in risk between housed and unhoused teenagers highlight the importance of understanding characteristics related to sexual health decision-making and sexual behavior for homeless adolescents, the consistent increase of undesirable outcomes underscores the urgency of the issue. Unsheltered youth are more likely to be sexually active and have a greater tendency to engage in risky sexual behaviors like multiple partnerships, survival sex, substance use surrounding sex, and inconsistent contraceptive use [2,6,12]. A propensity for homeless youth to participate in riskier sexual behaviors predisposes them to worse outcomes from the start. Behavior, and the biopsychosocial factors related to behavior, are undoubtedly related to reproductive health risks and outcomes in this population. Understanding these behaviors and/or their predictive factors is essential for improving reproductive health care and outcomes for unsheltered teenagers.

It is clear the homeless adolescent population experiences poor outcomes and is more likely to engage in risky sexual behaviors when compared to their housed peers. However, the biopsychosocial characteristics with significant relationships to sexual behavior are unknown. Many studies have focused on relationship, individual, educational, and contextual factors as they relate to sexual behaviors; most have focused on contraceptive use among homeless youths, at least during vaginal sex. Few studies though, have assessed if certain demographic or biopsychosocial characteristics predict consistent contraceptive use [2,6-21]. Even fewer have focused on characteristics that may indicate a higher risk for sexual activity. This study seeks to address this gap by testing the relationship between contraceptive use, sexual activity, and the presence of a diverse array of demographic and biopsychosocial factors including, but not limited to, age, race/ethnicity, history of suicide attempt, history of a traumatic event, and substance use. This study tested the hypothesis that biopsychosocial factors have significant relationships with sexual activity and contraceptive utilization among homeless adolescents. By better characterizing the impact certain factors may have on contraceptive utilization, informed interventions can be developed and implemented in spaces where homeless adolescents access healthcare, through both practice and policy. Our study aim was to determine whether biopsychosocial factors are associated with sexual activity and contraceptive utilization among homeless shelter adolescents.

This work was accepted for presentation, in part, as an abstract, titled “Relationship Between Sexual Activity, Contraceptive Utilization and Biopsychosocial Characteristics Among Homeless Adolescents.” at the North American Society of Pediatric and Adolescent Gynecology, March 18-20, 2021. 

## Materials and methods

A retrospective cohort study was conducted using clinical records of adolescents seen at a youth crisis housing shelter in Bethlehem, Pennsylvania between 2/19/2015 and 9/5/2019. Adolescents seen for state-mandated physical exams and/or acute care were included in the final analysis. Adolescents at the housing shelter who were not seen for a state-mandated physical exam and/or acute care were excluded from the study. Medical visits, for both state-mandated and acute care, were performed by a single hospital network. Demographic characteristics, biopsychosocial factors (e.g., childhood trauma, history of abuse, substance use history), sexual history, and contraceptive utilization were variables included in the extraction. Biopsychosocial variables recorded were obtained by patient self-report. Data collected for this study were de-identified and stored in a password-protected database to which only members of the study team had access. This study was approved by the Lehigh Valley Health Network Institutional Review Board (STUDY00000346).

Descriptive statistics were calculated to generate a summary of the demographic characteristics and reported medical, behavioral, social, and sexual history for the sample. For normally distributed continuous variables, the mean with standard deviation was reported. Categorical variables were presented as frequencies and percentages.

To test the hypothesis that certain biopsychosocial characteristics were associated with being sexually active and/or utilizing contraception, a Chi-square Test of Independence was conducted. Analyses were two-tailed and a p-value of <0.05 was considered statistically significant. Relative risk ratios (RR) were calculated for certain demographic and biopsychosocial characteristics. Multivariable logistic regression models, controlling for potential confounders identified in univariate analysis, were developed to evaluate the relationship between biopsychosocial characteristics and sexual activity, and adjusted odds ratios (AOR) with 95 percent confidence intervals (CI) were derived from these models. StataSE 16.0 software (STATA, College Station, Texas, USA) was used for statistical analysis.

## Results

The clinical records of 440 adolescents were found to meet the study inclusion criteria of having had a state-mandated physical exam and/or an acute care visit and were reviewed for sexual activity, contraceptive utilization, demographic and biopsychosocial data. The majority of this study population was female (n=245, 55.7%) and white (n= 71, 47.0%), though only 151 of the 440 had reported data on race. Ethnicity (Hispanic or Latino vs. “not” Hispanic or Latino) was reported in only eighty-eight records, the majority of which were Hispanic/Latino (n=70, 79.6%). The mean age of the population was 15.32 (SD=1.62) with a range from 11-20 years old (Table 1).

**Table 1:** Demographic, Sexual Activity, and Contraceptive Use Characteristics MtF - Male to female; FtM - Female to male

	Entire Sample (N=440)	Sexually Active (N=233)	Not Sexually Active (N=201)	Sexually Active With Contraception (N=148)	Sexually Active Without Contraception (N=79)
Age mean (SD)	15.32 (1.62)	15.85 (1.41)	14.69 (1.64)	15.95 (1.02)	15.77 (1.49)
Gender n(%)					
Male	187 (42.50)	93 (39.91)	92 (45.77)	68 (45.95)	22 (27.85)
Female	245 (55.68)	137 (58.80)	104 (51.74)	79 (53.38)	56 (70.89)
Transgender (MtF or FtM)	8 (1.82)	3 (1.29)	5 (2.49)	1 (0.68)	1 (1.27)
Race n(%)	(N=151)	(N=82)	(N=68)	(N=57)	(N=21)
Black/African American	55 (36.42)	32 (39.02)	22 (32.35)	20 (35.09)	9 (42.86)
White	71 (47.02)	38 (46.34)	33 (48.53)	28 (49.12)	10 (47.62)
Asian	3 (1.99)	1 (1.22)	2 (2.94)	1 (1.75)	0 (0.00)
Other	2 (1.32)	2 (2.44)	0 (0.00)	2 (3.51)	0 (0.00)
Multiracial	20 (13.25)	9 (10.98)	11 (16.18)	6 (10.53)	2 (9.52)
Ethnicity n(%)	(N=88)	(N=47)	(N=41)	(N=32)	(N=14)
Hispanic/Latino	70 (79.55)	38 (80.85)	32 (78.05)	26 (81.25)	12 (85.71)
Not Hispanic/Latino	18 (20.45)	9 (19.15)	9 (21.95)	6 (18.75)	2 (14.29)

Sexual activity

Among the 440 records included in the analysis, 53.0% (N=233) of adolescents reported being sexually active. Those adolescents were more often female (55.8%, N=137) and white (46.3%, N=38). Despite the frequency with which white female adolescents reported sexual activity, no statistically significant relationship of gender and/or race with sexual activity was identified. Age, however, did significantly relate to sexual activity; the mean age of sexually active adolescents was 15.8+1.4 years, whereas adolescents who denied sexual activity had a mean age of 14.7+1.6 years (p<0.001) (Table 2).

**Table 2:** Demographic Characteristics of Sexual Activity MtF - Male to female; FtM - Female to male

	Entire Sample (N=440)	Sexually Active (N=233)	Not Sexually Active (N=201)	P Value
Age mean (SD)	15.32 (1.62)	15.85 (1.41)	14.69 (1.64)	<0.001
Gender n(%)				0.211
Male	187 (42.50)	93 (39.91)	92 (45.77)	
Female	245 (55.68)	137 (58.80)	104 (51.74)	
Transgender (MtF or FtM)	8 (1.82)	3 (1.29)	5 (2.49)	
Race n(%)	(N=151)	(N=82)	(N=68)	0.499
Black/African American	55 (36.42)	32 (39.02)	22 (32.35)	
White	71 (47.02)	38 (46.34)	33 (48.53)	
Asian	3 (1.99)	1 (1.22)	2 (2.94)	
Other	2 (1.32)	2 (2.44)	0 (0.00)	
Multiracial	20 (13.25)	9 (10.98)	11 (16.18)	
Ethnicity n(%)	(N=88)	(N=47)	(N=41)	0.952
Hispanic/Latino	70 (79.55)	38 (80.85)	32 (78.05)	
Not Hispanic/Latino	18 (20.45)	9 (19.15)	9 (21.95)	

Other behavioral, social, and psychiatric elements of patient histories were found to be related to sexual activity in this population (Table 3). Those adolescents who reported suicidal thoughts, suicide attempts, and/or self-harm behaviors more than six months prior to their encounter were more likely to be sexually active (RR 1.23 [95% CI 1.03-1.46]; p=0.02). Engagement in aggressive or violent behavior, either in remote or recent past, also increased the risk of being sexually active (RR 1.21 [95% CI 1.01-1.46]; p=0.04). A relationship between the presence of a traumatic event in an adolescent’s history and being sexually active was identified as well (RR 1.24 [95% CI 1.04-1.48]; p=0.03). Social factors, such as the use of tobacco, alcohol, marijuana, and other drugs drastically increased the likelihood that an adolescent was sexually active (RR 2.27 [95%CI 1.86-2.77]; p<0.001). Finally, the presence of anxiety or attention-deficit/hyperactivity disorder (ADHD) was related to sexual activity (p<0.001). Multivariable logistic regression models, controlling for potential confounders, were developed to evaluate the relationship between biopsychosocial characteristics and sexual activity, and adjusted odds ratios (AOR) with 95 percent confidence intervals (CI) were derived from these models. After controlling for confounders, including age, history of suicidal thoughts, suicide attempts, and/or self-harm behaviors, aggressive or violent behavior (remote or recent past), history of trauma, substance use, and anxiety or ADHD, older age and substance use remained significantly associated with sexual activity (AOR 1.58 [95% CI 1.36-1.84]; p<0.001 and AOR 5.09 [95% CI 3.22-8.05]; p<0.001, respectively).

**Table 3:** Relationship of Biopsychosocial Factors to Sexual Activity HPV - Human papillomavirus; PTSD - Post-traumatic stress disorder; ADHD - Attention-deficit/hyperactivity disorder; Psych - Psychiatric

	Entire Sample (N=440)	Sexually Active (N=233)	Not Sexually Active (N=201)	P Value
Immunization History n(%)		(N=149)	(N=139)	
HPV		46 (30.87)	42 (30.22)	1.000
Behavioral History n(%)				
Suicide/Self Harm Behaviors Present in last 6 months	97 (22.35)	50 (21.46)	46 (22.89)	0.810
Suicide/Self Harm Behaviors Present more than 6 months ago	170 (39.35)	103 (44.21)	66 (32.84)	0.020
Suicide/Self Harm Behaviors Present Ever	194 (44.80)	111 (47.64)	82 (40.80)	0.182
Aggressive or Violent Behavior Present in the last 6 months	156 (35.94)	94 (40.34)	62 (30.85)	0.050
Aggressive or Violent Behavior Present more than 6 months ago	226 (52.19)	131 (56.22)	95 (47.26)	0.077
Aggressive or Violent Behavior Present Ever	254 (58.53)	147 (63.09)	107 (53.23)	0.048
History of Traumatic Event	114 (26.27)	71 (30.47)	42 (20.90)	0.031
Social History n(%)				
Tobacco	120 (27.65)	106 (45.49)	14 (6.97)	<0.001
Alcohol	52 (12.01)	45 (19.31)	7 (3.48)	<0.001
Marijuana	163 (37.56)	124 (53.22)	39 (19.40)	<0.001
Other Drugs	42 (9.68)	38 (16.31)	4 (1.99)	<0.001
Psychiatric History n(%)				
Depression	132 (30.00)	78 (33.48)	53 (26.37)	0.133
Bipolar	49 (11.14)	13 (5.58)	17 (8.46)	0.323
PTSD	26 (5.91)	4 (1.72)	9 (4.48)	0.161
Anxiety	72 (16.36)	4 (1.72)	30 (14.93)	<0.001
Psychotic Disorder	3 (0.68)	3 (1.29)	0 (0.00)	0.301
ADHD	104 (23.64)	15 (6.44)	56 (27.86)	<0.001
Other	63 (14.32)	9 (3.86)	28 (13.9)	<0.001
>1 Psych Diagnosis	121 (27.50)	70 (30.04)	45 (22.39)	0.091
None	196 (44.55)	104 (44.64)	92 (45.77)	0.888

Contraception utilization

Of those 233 who reported sexual activity, 63.5% (N=148) reported utilization of some form of contraception. Adolescents who did endorse the use of contraception tended to be female (53.4%, N=79), white (49.1%, N=57) and had a mean age of 15.95+1.02 years (Table 4). A significant difference existed between female and male adolescents, with female adolescents being far more likely than their male peers to use contraception (p=0.013). Despite the majority of contraceptive users being white and/or older, neither race nor age was found to be significantly related to the use of contraception.

**Table 4:** Demographic Characteristics of Contraceptive Utilization MtF - Male to female; FtM - Female to male

	Entire Sample (N=440)	Sexually Active With Contraception (N=148)	Not Sexually Active Without Contraception (N=79)	P Value
Age mean (SD)	15.32 (1.62)	15.95 (1.02)	15.77 (1.49)	0.287
Gender n(%)				0.013
Male	187 (42.50)	68 (45.95)	22 (27.85)	
Female	245 (55.68)	79 (53.38)	56 (70.89)	
Transgender (MtF or FtM)	8 (1.82)	1 (0.68)	1 (1.27)	
Race n(%)	(N=151)	(N=57)	(N=21)	0.715
Black/African American	55 (36.42)	20 (35.09)	9 (42.86)	
White	71 (47.02)	28 (49.12)	10 (47.62)	
Asian	3 (1.99)	1 (1.75)	0 (0.00)	
Other	2 (1.32)	2 (3.51)	0 (0.00)	
Multiracial	20 (13.25)	6 (10.53)	2 (9.52)	
Ethnicity n(%)	(N=88)	(N=32)	(N=14)	1.000
Hispanic/Latino	70 (79.55)	26 (81.25)	12 (85.71)	
Not Hispanic/Latino	18 (20.45)	6 (18.75)	2 (14.29)	

## Discussion

Compared to their housed peers, unsheltered adolescents are more likely to engage in risky sexual behaviors and to experience negative reproductive health outcomes. This single-site study examined the relationship that certain demographic, behavioral, psychiatric, and social factors have on sexual activity and contraceptive utilization among sheltered adolescents. The association of older age with sexual activity was expected. However, it is important to note that the mean age reported is reflective of the adolescent’s age at the time of their encounter, not of their age at sexual initiation. As such, younger adolescents may still be at risk for becoming sexually active and may be a population that could benefit from targeted prevention interventions and/or education. Further, this could have implications for incorporating screening tools like Home, Education/employment, peer group Activities, Drugs, Sexuality, and Suicide/depression (HEADSS) into preventive and acute care visits for younger populations if they are experiencing housing instability.

Reassuringly, in this study sample, 63.5% of adolescents who were sexually active reported using some form of contraception. However, specific types of contraception utilized and the frequency with which those forms were used were not reported. Gender was a good predictor of contraceptive use, with 53.4% of those reporting contraception use being female. It is likely that the higher propensity of sexually active females to utilize some form of contraception when compared to their sexually active male peers is, in part, related to the cultural and social pressures that place the responsibility of contraception on the female partner in heterosexual relationships. Resources exist for young women and those with female reproductive anatomy that do not exist for young men, especially regarding access to birth control, condoms, options counseling, and other reproductive health services. The emphasis on social programming that prioritizes the reproductive health of women may also be a factor underlying the relationship between gender and contraception. Regardless of the social structures that influence this association, it is demonstrative that young men stand to benefit from focused reproductive health interventions, as they are not immune to the consequences their female peers face (e.g., pregnancy) [22]. The psychiatric and behavioral factors assessed (listed in the methods section) did not appear to relate to contraceptive use.

Certain mental and behavioral health characteristics were found to have relationships with sexual activity. A history of aggressive behavior, the experience of a traumatic event, or a diagnosis of anxiety all increased the likelihood that an adolescent would be sexually active. These are not uncommon in the homeless adolescent population. Youth who reported a remote history of self-injurious behavior, suicidal ideation, and/or suicide attempt were more likely to be sexually active. It is possible that homeless adolescents who engage in self-harm behaviors lack robust social support and/or coping mechanisms when compared to homeless youth who do not have a self-harm history. Perceivably, adolescents are more likely to have better mental and sexual health outcomes if they experience peer connectedness and hope for the future. As such, the gaps in social structure and resources for coping among those who engage in self-harm likely exacerbate their sexual risk-taking behaviors. There is also the possibility that sexual activity is another manifestation of their self-injurious behaviors (SIB); the notion of sex as self-injury (SASI) is relatively new and understudied [23,24]. Those who engage in SASI report that it feels compulsive and addictive, and is associated with ever-increasing risk-taking behaviors, often including deliberate sexual violence [24]. While not all those who engage in SASI also engage in more advert SIB, they do often coincide [23]. It is possible that this is a reason for the connection between SIB and suicidal ideation/attempts, and the sexual activity of those in this study, but further research would be needed.

A very clear relationship between substance use and sexual activity was delineated, which is concerning given the risk for substance use among homeless sheltered youth at baseline. This relationship remained significant even when examined at the level of the substance used (tobacco, alcohol, marijuana, and/or other drugs). Adolescents in this sample were five times more likely to be sexually active if they were using substances. However, drug and alcohol use can precede or proceed with sexual activity, and the influence that substance use ultimately has on sexual behavior and decision-making is unclear. Additionally, the amount and frequency of substance use were not extracted for this study, making it difficult to determine if heavy users had behavior that differed from more recreational users.

Existing literature further characterizes the sexual behaviors that are associated with substance use and has shown connections to both sexual risk behaviors and sexual victimization in this population [25]. Sexual risk behaviors typically refer to the exchanging of sexual services for things such as shelter, food, money, drugs, or alcohol while sexual victimization typically refers to the threat or experience of sexual harm or nonconsensual sexual handling [25]. These are important aspects of homeless adolescents’ social histories to be assessed during encounters, as teenagers may not all be forthcoming about taboo behaviors, nor may they be forthcoming about instances of abuse. Special attention should be paid to those who use, or unsafely use, substances in this population to evaluate their risk of negative reproductive outcomes based on the tendency to engage in risky behaviors and the risk of sexual victimization. The finding that substance use has a relationship with sexual activity also underscores the dire necessity for better access to substance use care in this population. Addiction care is notoriously hard to access, cover, and utilize effectively, and improvements could have implications for both mental and reproductive health in this population.

A major limitation of this study was the exclusion of many charts during the data extraction process due to incomplete documentation. Given that this was a retrospective cohort study, which relied on chart review for data, by nature the project was limited by the data available for analysis. As a consequence, inferences based on information such as the form of contraception, duration, frequency, or amount of drug use, and engagement in sexual risk behaviors (e.g., sex work, survival sex, street prostitution) could not be made. Psychiatric, medical, social, and sexual histories were provided by clients of the shelter or their parents, which allowed for recall bias. Additionally, the sample was derived from a single site, which limits the generalizability. Differences outside of the catchment area of the shelter would not have been accounted for in the analysis, and youth experiencing homelessness in other areas may not share the same common risk factors as those in the study sample. The youth cared for by this shelter, and as a result, included in this study experienced homelessness to varying lengths and degrees. The heterogeneity of individual experiences with homelessness among these adolescents also presents a limitation. Not all experiences of homelessness are equal in length, severity, character, and the variability present in this study sample may differ from that of another portion of the homeless adolescent population. The final limitation that bears highlighting is the difficulty in applying conclusions to the LGBTQ (lesbian, gay, bisexual, transgender, queer) population. Despite the fact that nearly 40% of homeless adolescents identify as LGBTQ+ [22], this sample had an extraordinarily small proportion of participants who identified as transgender (1.8%, N= 8), and the forms used in the clinical setting did not collect data regarding sexual orientation (e.g., lesbian, bisexual, gay, asexual, pansexual, demisexual). Consequently, conclusions regarding the relationship of sexual orientation and a transgender or nonbinary gender identity with sexual behavior cannot be drawn from this study.

Several areas for focus, attention, intervention, and education have been illuminated by the deduced relationships biopsychosocial characteristics have with sexual activity and contraceptive use. It is important to note that typical avenues for sexual and reproductive health education may not be accessible to homeless youth. Sex education in the United States is delivered primarily via the public education system, but many homeless adolescents struggle to enroll in and complete their primary and secondary education [25]. In the case of homeless youth, it is important to consider the resources to which they do and do not have access when developing and implementing interventions. Bringing interventions directly to the spaces where homeless youth and healthcare interact may catch those who fall through the cracks simply because they lack access to the conventional resources.

Future research based on these observations can inform inquiry into how these relationships impact outcomes or how they may be leveraged for quality improvements. These findings indicate an opportunity for efforts, moving forward, to improve sexual health outcomes in older teenagers who report using illicit or recreational substances and to make healthcare interventions the most effective for this population.

## Conclusions

In our single shelter study, adolescents with housing instability face risks for poor reproductive health outcomes. Females self-reported sexual activity using contraception more than males. This study confirmed that characteristics within the sheltered homeless youth population were related to sexual activity and contraceptive use. Substance use and older age remained predictors of sexual activity when controlled for confounders. After adjustment, older age and substance use were associated with sexual activity. By better understanding the impact these factors can have on contraceptive utilization, informed policy and practice interventions can be developed and implemented in spaces where homeless adolescents access healthcare in a way that will have the strongest impact.
